# Course and treatment of severe osteoporosis complicated by calciphylaxis: a case report

**DOI:** 10.1093/jbmrpl/ziae154

**Published:** 2024-11-22

**Authors:** Ayako Tominaga, Keiji Wada, Yoshiharu Kato, Ken Okazaki

**Affiliations:** Department of Orthopedic Surgery, Tokyo Women’s Medical University, Tokyo, 162-8666, Japan; Department of Orthopedic Surgery, Tokyo Women’s Medical University, Tokyo, 162-8666, Japan; Department of Orthopedic Surgery, Kita Shinagawa 3rd Hospital, Tokyo, 140-0001, Japan; Department of Orthopedic Surgery, Tokyo Women’s Medical University, Tokyo, 162-8666, Japan

**Keywords:** calciphylaxis, osteoporosis, vertebral fracture, calcium, denosumab

## Abstract

Calciphylaxis, also known as calcific uremic arteriolopathy (CUA), is a rare disorder with many unknown treatment and diagnostic aspects. It is characterized by calcification and thrombosis of small blood vessels. This disease leads to progressive skin calcification, necrotizing ulcers, and infections and is associated with a high mortality rate. Although primarily affected sites tend to be on skin, those affecting bones are also significant. We report a case of CUA complicated with rapidly progressing multiple vertebral fractures and severe osteoporosis. The patient experienced a series of five vertebral fractures within 5 months after hospitalization, and blood tests revealed abnormally high levels of bone resorption marker bone-type tartrate-resistant acid phosphatase (TRACP-5b). Consequently, intravenous sodium thiosulfate and hyperbaric oxygen therapy were administered for the treatment of skin lesions caused by calciphylaxis, and brace therapy and denosumab treatment were initiated for vertebral fractures. This approach rapidly decreased TRACP-5b levels and arrested the chain of vertebral fractures. We concluded that to maintain the quality of life of patients with CUA, early treatment of primary skin lesions as well as comorbid conditions is essential.

## Graphical Abstract

**Graphical Abstract:**
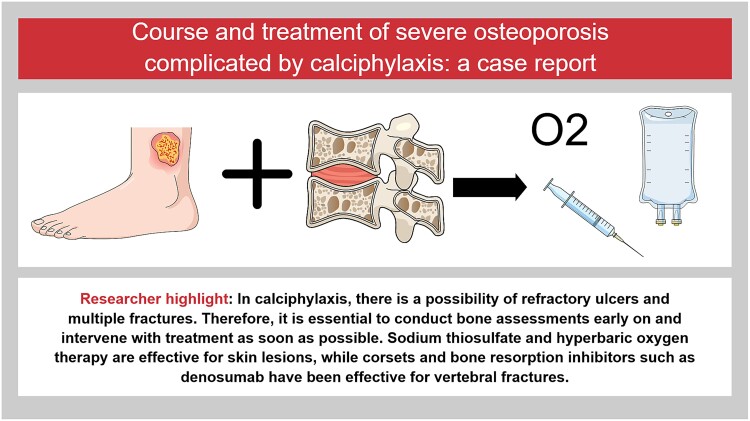

## Introduction

Calciphylaxis, also known as calcific uremic arteriolopathy (CUA), is a rare disorder observed in patients with end-stage renal disease (ESRD).^1–3^ However, it can occur outside ESRD and has been reported in patients administered with warfarin or teriparatide.^2^^,^^3–7^ The etiology of CUA remains unclear; however, CUA has been strongly associated with microvascular calcification and thrombus formation.^1–3^ This disease induces progressive skin calcification, necrotic ulcers, and infection, and it is associated with a high mortality rate.^1–3^ Although most attention is given to skin as the primary site, bones are also significantly affected. We report a case of multiple vertebral fractures and severe osteoporosis in a patient with CUA in which the need for attention to osteoporosis in CUA treatment is emphasized.

## Case description

A 41-yr-old woman presented to our hospital with systemic lupus erythematosus (SLE), which started in 1995; secondary phospholipid antibody syndrome (APS), which started in 2009; and stroke without sequelae in 2010, 2012, and 2017. The patient was administered warfarin (2.5 mg) and cilostazol (100 mg) for APS and tacrolimus hydrate (2.5 mg) and prednisolone (10 mg) for SLE.

**Figure 1:**
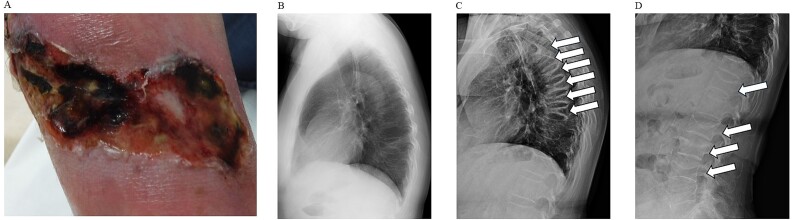
(A) The skin lesions caused by calciphylaxis. Multiple painful skin ulcers on the anterior part of the lower right leg were resistant to treatment. (B) One month after admission, the patient experienced a fracture of the ninth thoracic vertebra. Arrows indicate the fracture site. (C) Five months after admission, the patient sustained six new vertebral fractures. All fractures were in the thoracic spine. (D) The eighth dose of denosumab was delayed, and the patient had thoracic and lumbar vertebral fractures that appeared to be a rebound from the denosumab.

In June 2019, the patient presented to the Dermatology Department with multiple painful skin ulcers on the anterior part of the right lower leg that did not improve (Figure 1A). A skin biopsy was performed, and histopathology confirmed the diagnosis of a suspected thrombus in the upper dermis along with a ring of calcified deposits in the subdermis and small-to-medium arteries of the adipose tissue. The patient was diagnosed with CUA and admitted to our hospital in June. The skin biopsy area was also exacerbated, consistent with the diagnosis of CUA.

After admission, antibiotics were administered, warfarin was replaced with heparin, and steroids were tapered off. In July, intravenous sodium thiosulfate (10 g/d^*^3/wk) was initiated, and hyperbaric oxygen therapy was performed. In November, skin epithelialization progressed, and the patient was discharged from the hospital and continued outpatient treatment. In December, the skin ulcers improved.

## Osteoporosis

### Description of vertebral fractures and the osteoporosis process

The patient had been taking steroids for a long time and was prescribed an active Vitamin D supplement (eldecalcitol, 0.75 μg) by another department. Furthermore, the patient had an 11th thoracic vertebra (Th11) fracture of unknown onset. In late August 2019, while hospitalized, the patient experienced lateral back pain without a specific trigger and was referred to an orthopedic surgeon, who performed a simple radiographic examination and found a Th9 fracture (Figure 1B). The patient was treated with braces. The patient had a Th8 fracture without any trigger in late September, a Th7 fracture in late October, and Th4, Th5, and Th6 vertebral fractures in late November (Figure 1C). Blood tests revealed elevated levels of the bone resorption marker bone-type tartrate-resistant acid phosphatase (TRACP-5b); thus, denosumab therapy was initiated in November 2019. Serum-corrected calcium (Ca), phosphorus (P), intact type 1 amino-terminal propeptide (P1NP), and TRACP-5b levels promptly decreased after treatment onset. However, no evidence of hypocalcemia (<8.8 mg/dL) or hypercalcemia (>10.5 mg/dL) was observed during the study.^8^^,^^9^

High corrected Ca levels are a risk factor for CUA; however, they did not exceed normal levels during the disease. After the initiation of denosumab, the patient no longer experienced fractures. In general, denosumab is administered once every 6 months; however, for tooth extraction in 2023, denosumab’s administration was discontinued for approximately 2 months. Hence, it was administered in the eighth month. This delay caused a rebound fracture, resulting in Th12, L2, L3, and L4 fractures (Figure 1D). We continued treatment with zoledronic acid, which has a lower rebound potential, and bone density tests were performed (spine BMD (Figure 2-1A) and total hip BMD (Figure 2-1B)). Blood test results (corrected Ca (Figure 2-2A), P (Figure 2-2B), estimated glomerular filtration rate (eGFR) (Figure 2-2C), P1NP (Figure 2-2D), TRACP-5b (Figure 2-2E), and intact parathyroid hormone (intact PTH) (Figure 2-2F)) are shown in the graphs.

**Figure 2-1:**
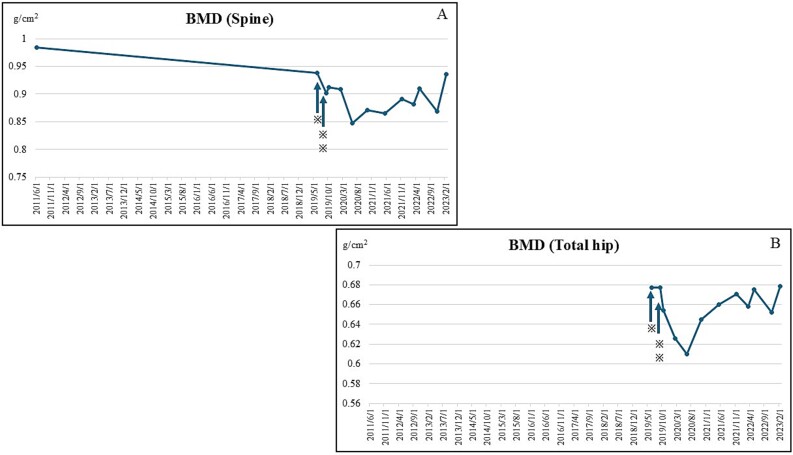
(A) Spinal BMD levels decreased rapidly after hospitalization; however, they gradually increased after the second denosumab inoculation. (B) Hip BMD levels decreased rapidly after hospitalization; however, they gradually increased after the second denosumab inoculation. An asterisk indicates the date of admission, and a double asterisk shows the date of the first denosumab implementation. ^*^indicates the admission date, and ^*^^*^indicates the administration of denosumab.

**Figure 2-2:**
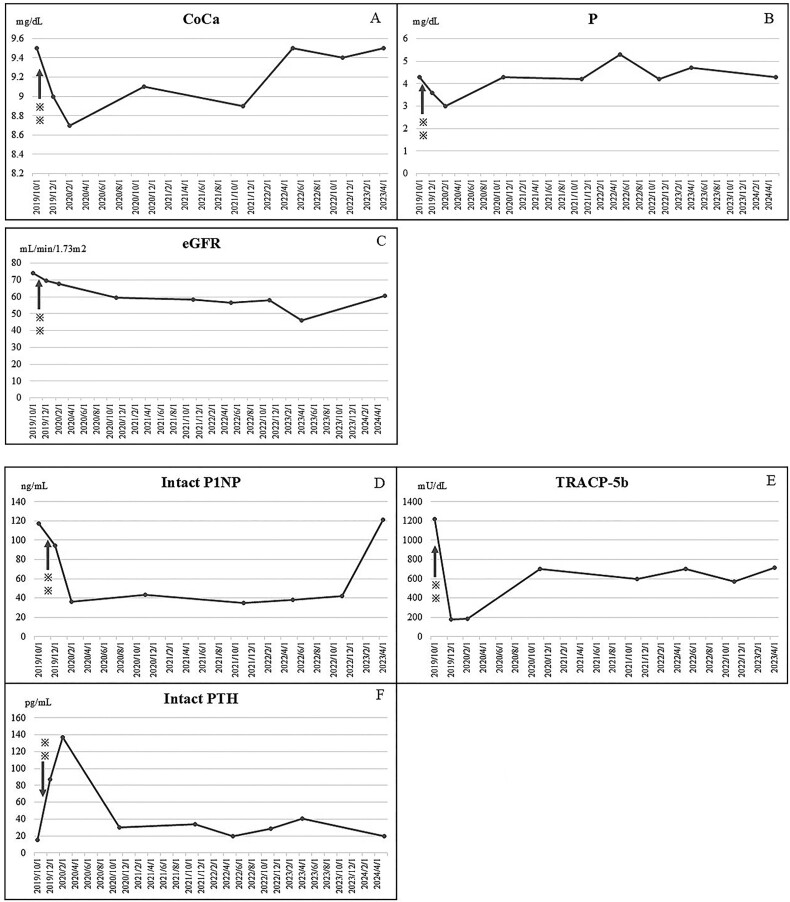
(A) A decrease in corrected calcium levels was detected after the initiation of denosumab treatment. However, no evidence of hypo- or hypercalcemia was observed during the study. (B) Blood phosphorus (P) levels decreased after denosumab administration but returned to baseline after about 1 yr. (C) Estimated glomerular filtration rate (eGFR) gradually declined, but there was no rapid deterioration during the course. There was a temporary aggravation in April 2023, but it later recovered. (D) Intact type 1 amino-terminal propeptide (intact P1NP) levels decreased rapidly after denosumab initiation and remained low after that. (E) Bone-type tartrate-resistant acid phosphatase (TRACP-5b) levels decreased promptly after denosumab initiation but then increased again. However, they did not return to baseline. (F) Intact parathyroid hormone (intact PTH) increased inversely with the decrease in blood calcium levels after denosumab administration, but later returned to baseline. ^*^^*^indicates the administration of denosumab.

## Discussion

CUA is a rare refractory disease characterized by microvascular calcification and thrombus formation.^1–3^ Although it is most commonly reported in patients with ESRD, CUA has been reported in patients taking teriparatide or warfarin, as in this case.^2^^,^^3–7^ Although skin lesions are considered primary, we have experienced patients with CUA manifesting with severe osteoporosis. Cases of osteoporosis associated with CUA have not been reported, and its treatment targeting BMD and bone metabolic markers makes this report valuable.

CUA was first described by Dr. Seyle in 1961 after conducting an experiment inducing systemic subcutaneous tissue calcification in rats.^2^ Although the number of clinical reports has increased since the 1960s, the prevalence of the disease has been reported to significantly vary according to different races and countries.^2^^,^^3^ The incidence in the United States has been shown to be 3.49 cases per 1000 patients; the annual incidence in Germany has been reported to be approximately 0.04%; in Japan, it is less than three cases per 10 000 patients; and in Australia, 47 cases have been reported over 5 yr.^3^ Clinical findings include skin lesions, accompanied by severe pain that progresses to ulcers and black necrosis. Calcification of blood vessels in other organs and tissues, such as skeletal muscle and lungs, has also been reported.^1–3^^,^^5^ Treatment approaches include removal of risk factors, wound care, pain management, intravenous sodium thiosulfate, Vitamin K supplementation, bisphosphonate administration, antibiotics, and parathyroidectomy. If necessary, anticoagulants, kidney transplantation, and hyperbaric oxygen therapy should be considered.^1–3^ Despite these measures, the prognosis remains very poor, with an annual mortality rate reported to be 40%-80%.^1–3^

The exact etiology of CUA is not fully understood; however, vascular wall calcification and thrombosis have been identified as major components of the pathological process associated with CUA, and they are believed to involve imbalances in calcium and phosphorus metabolism.^2^ It has been shown that the same factors that cause bone demineralization in osteoporosis contribute to vascular calcification, making it conceivable that osteoporosis may be associated with CUA.^8^^,^^9^ Genes, proteins, cytokines, and transcription factors required for bone development and maintenance have also been shown to mediate vascular calcification.^10^^,^^11^ In SLE, multiple organs are affected because of inflammatory autoimmune disease.^12^ Inflammation promotes atherosclerosis, and its association with calciphylaxis has also been reported.^12^ Cytokines, such as IL-6 and TNF-α, facilitate atherosclerosis, and vascular damage activates cells like osteoblasts and osteoclasts, further accelerating atherosclerosis as reported in some studies.^12^^,^^13^ There are also studies reporting that B lymphocytes from SLE patients induce bone resorptive activity.^12^^,^^14^ However, many aspects of osteoporosis induced by CUA and its pathophysiology remain unclear.

Furthermore, sodium thiosulfate, used for treating CUA in a foundational study involving rats, has been reported to promote urinary calcium excretion and induce a negative calcium balance, thus potentially preventing vascular calcification.^15^ However, bone strength also decreased, suggesting that sodium thiosulfate intensifies osteoporosis, not only CUA.^15^ Bisphosphonates have been used for treating CUA because they are believed to be effective because of their potential to interfere with arterial calcification.^16^ In particular, it has been shown that 200 mg per day for 14 d is effective.^16^ The bisphosphonate treatment described in these studies is not primarily intended to treat osteoporosis. In this case, as orthopedic surgeons, we intervened with treatment mainly to suppress osteoclast activity and stop the cascade of vertebral fractures, caused by a rapid decline in BMD, the series of vertebral fractures occurring in a short period, and abnormally high TRACP-5b levels. Oral bisphosphonates have been shown to take 6-12 months to become effective; therefore, we used denosumab, which can suppress bone resorption within a shorter period.^17^ Although the use of romosozumab had been considered, it was not selected because of cerebral infarctions during the patient’s anamnesis. Teriparatide was also not used because it carries the risk of CUA. After initiating denosumab therapy in this patient, a rapid decrease in P1NP and TRACP-5b levels was detected, and the ongoing cascade of vertebral fractures was halted. After the initiation of denosumab therapy, the Ca and P levels improved rapidly, suggesting a positive effect on the pathology of calciphylaxis. However, although P1NP levels remained low during the continuation of denosumab administration, TRACP-5b levels fluctuated between 600 and 800 mU/dL, although they were lower than those observed before treatment. The clinical course after starting denosumab was uneventful; however, in 2023, denosumab administration was discontinued for 2 months due to dental treatment. Thus, multiple vertebral fractures, likely due to denosumab rebound, occurred.^18^ In such cases in which the disease is aggravated by various comorbidities, the risk of unexpected delays in denosumab treatment is high. Therefore, preventing the incidental interruptions of denosumab or considering switching to long-acting modes of treatment, such as zoledronic acid infusions, is necessary. Conversely, in this case of CUA, the TRACP-5b level before treatment was 1220 mU/dL, indicating high bone turnover. However, it has been reported that bone lesions associated with CUA may show low bone turnover in some cases. In such cases, osteoporosis treatment with different medications may be required.^14^^,^^19^ When treating bone metabolic abnormalities associated with CUA, it is necessary to determine whether bone turnover is elevated or reduced.

## Conclusion

CUA is a rare refractory disease with a high mortality rate; however, early management of comorbidities is required to maintain a higher quality of life after disease remission. Osteoporosis and vertebral fractures are thought to be induced by CUA and sodium thiosulfate administration. An appropriate bone resorption inhibitor should be selected based on clinical symptoms, bone density tests, and changes in bone metabolism markers are necessary. We could quickly reduce TRACP-5b levels and stop the repeated occurrence of vertebral fractures using denosumab. However, being cautious regarding the rebound effects exerted by denosumab is important, and considering switching osteoporosis treatment schemes at an appropriate time is also necessary.

## Data Availability

The data underlying this article will be shared upon reasonable request to the corresponding author.
